# Association of Circulating Osteoprotegerin Level with Blood Pressure Variability in Patients with Chronic Kidney Disease

**DOI:** 10.3390/jcm11010178

**Published:** 2021-12-29

**Authors:** Sang Heon Suh, Tae Ryom Oh, Hong Sang Choi, Chang Seong Kim, Kook-Hwan Oh, Joongyub Lee, Yun Kyu Oh, Ji Yong Jung, Kyu Hun Choi, Seong Kwon Ma, Eun Hui Bae, Soo Wan Kim, on behalf of the Korean Cohort Study for Outcomes in Patients with Chronic Kidney Disease (KNOW-CKD) Investigators

**Affiliations:** 1 Department of Internal Medicine, Chonnam National University Medical School and Chonnam National University Hospital, Gwangju 61469, Korea; medssh1984@gmail.com (S.H.S.); tryeomoh@hanmail.net (T.R.O.); hongsang38@hanmail.net (H.S.C.); laminion@hanmail.net (C.S.K.); drmsk@hanmail.net (S.K.M.); 2 Department of Internal Medicine, Seoul National University Hospital, Seoul 03080, Korea; ohchris@hanmail.net; 3 Department of Prevention and Management, School of Medicine, Inha University, Incheon 22212, Korea; tp240@naver.com; 4 Department of Internal Medicine, Seoul National University, Seoul 08826, Korea; yoonkyuoh@gmail.com; 5 Department of Internal Medicine, Division of Nephrology, Gachon University of Gil Medical Center, Incheon 21565, Korea; jyjung@gachon.ac.kr; 6 Department of Internal Medicine, Institute of Kidney Disease Research, College of Medicine, Yonsei University, Seoul 03722, Korea; Khchoi6@yuhs.ac

**Keywords:** blood pressure variability, chronic kidney disease, osteoprotegerin

## Abstract

Circulating osteoprotegerin (OPG) is a biomarker for cardiovascular complications that are closely related to chronic kidney disease (CKD). To investigate the association between circulating OPG level with long-term visit-to-visit blood pressure variability (BPV) in patients with pre-dialysis CKD, a total of 1855 subjects with CKD from stage 1 to pre-dialysis stage 5 from a prospective cohort were analyzed. Long-term visit-to-visit BPV was determined by average real variability (ARV), standard deviation (SD), and coefficient of variation (CoV) of systolic and diastolic blood pressure (SBP and DBP). ARV of SBP (Adjusted β coefficient 0.143, 95% confidence interval 0.021 to 0.264) was significantly associated with serum OPG level. Although SD and CoV of SBP were not significantly associated with serum OPG level in multivariate linear regression analyses, restricted cubic spline visualized the linear correlation of serum OPG level with all of ARV, SD, and CoV. The association between serum OPG level and DBP variability was not significant. Subgroup analyses revealed that the association of serum OPG with BPV is more prominent in the subjects with Charlson comorbidity index ≤3 and in the subjects without history of diabetes mellitus. In conclusion, circulating OPG level is potentially associated with long-term visit-to-visit BPV in patients with pre-dialysis CKD.

## 1. Introduction

Blood pressure variability (BPV) is an emerging surrogate of cardiovascular (CV) outcomes, as it predicts the risk of CV events and all-cause mortality in general population [1,2,3], independent of mean blood pressure (BP). Among patients with chronic kidney disease (CKD), long-term visit-to-visit BPV is also associated with adverse CV outcomes [4]. Further, visit-to-visit BPV increases the risk of incident CKD in hypertensive individuals [5] and accelerates the decline of renal function in patients with CKD [6,7,8]. Provided that CV events are the leading cause of death in patients with reduced kidney function [9], the prediction of long-term BPV is becoming more and more important in the management of CKD patients.

Osteoprotegerin (OPG) is a decoy receptor of receptor activator of nuclear factor kappa-B ligand (RANKL) [10], which belongs to soluble tumor necrosis factor superfamily receptor [11]. OPG inhibits RANKL-mediated differentiation of osteoclast as well as activation and survival of mature osteoclasts [11], playing a pivotal role in the regulation of bone turnover. Circulating OPG levels increase with initiation of atherogenic diet, while exogenous OPG injection inhibits vascular calcification [12,13,14], indicating circulating OPG as a biomarker, but not a mediator, of atherosclerosis that is also closely related to CKD. Previous studies demonstrated that high serum OPG level is associated with the presence [15] and severity [16] of coronary artery calcification in patients with CKD. In this regard, elevated circulating OPG level has been proposed as a predictor of cardiovascular [17,18] and all-cause [19,20] mortality in patients with CKD. Serum OPG level is positively associated with peripheral artery disease, arterial stiffness, and arterial calcification in patients with end-stage renal disease [21,22,23,24]. Moreover, kidney transplantation decreases circulating level of OPG [25], and early post-transplantation circulating OPG level in serum predicts long-term patient survival up to 8 years [26], collectively suggesting a prognostic impact of circulating OPG level in patients with renal insufficiency. Nevertheless, the association between circulating OPG level and BPV has not been validated yet, especially in patients with CKD.

We here investigated the association between circulating OPG level with long-term visit-to-visit BPV in patients with pre-dialysis CKD. Taking advantage of various BPV indices, we intensively examined the linear correlation between serum OPG level and BPV. We also conducted subgroup analyses to address whether the association of serum OPG level with BPV modified by clinical contexts.

## 2. Materials and Methods

### 2.1. Study Design

**Figure 1:**
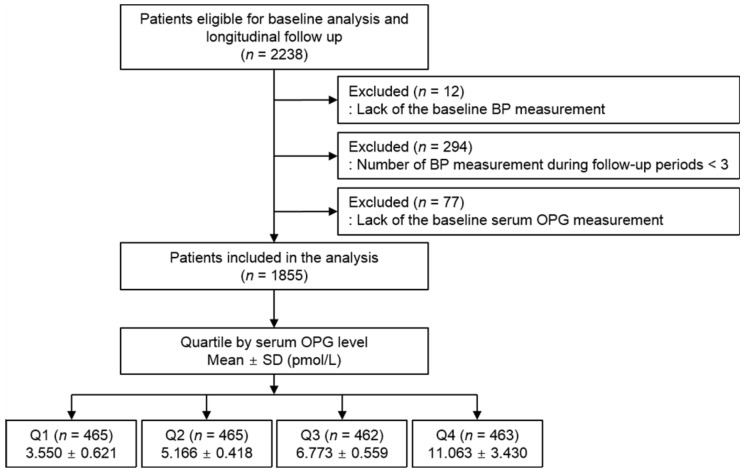
Flow diagram of the study participants. Abbreviations: ASV, average successive variability; BP, blood pressure; OPG, osteoprotegerin; Q1, 1st quartile; Q2, 2nd quartile; Q3, 3rd quartile; Q4, 4th quartile; SD, standard deviation.

The Korean Cohort Study for Outcomes in Patients with Chronic Kidney Disease (KNOW-CKD) is a nationwide prospective cohort study involving 9 tertiary-care general hospitals in Korea [27]. Korean patients with CKD from stage 1 to pre-dialysis stage 5, who voluntarily provided informed consent were enrolled. The study was conducted in accordance with the principles of the Declaration of Helsinki, and the study protocol was approved by the institutional review boards of participating centers. A total of 2238 subjects were longitudinally followed up. (Figure 1). After excluding those lacking the baseline measurement of BP, those with the number of BP measurement during follow-up periods less than three time, and those lacking the baseline measurement of serum OPG level, a total of 1855 subjects were finally included for the analyses. The median follow-up duration was 6.124 years.

### 2.2. Data Collection from Participants

Demographic information was collected from all eligible participants, including age, gender, comorbid conditions, and medication history (angiotensin-converting enzyme inhibitor/angiotensin II receptor blockers (ACEi/ARBs), diuretics, total number of antihypertensive drugs, statins). Trained staff members measured the height and weight of study participants. Body mass index (BMI) was calculated as weight divided by height squared. Venous samples were collected following overnight fasting, to determine hemoglobin, albumin, total cholesterol, low density lipoprotein cholesterol (LDL-C) high density lipoprotein cholesterol (HDL-C), triglyceride (TG), fasting glucose, high-sensitivity C-reactive protein (hs-CRP), 25-hydroxyvitamin D (25(OH) vitamin D) and creatinine levels at the baseline. Estimated glomerular filtration rate (eGFR) was calculated by Chronic Kidney Disease Epidemiology Collaboration (CKD-EPI) equation [28]. Urine albumin-to-creatinine ratio was measured in random, preferably second-voided, spot urine samples. Twenty-four-hour urine protein excretion was also determined.

### 2.3. Measurement of Serum OPG Concentration

Serum OPG level was measured by using enzyme-linked immunosorbent assay kit (BioVendor R&D, Brno, The Czech Republic) in the central laboratory (Lab Genomics, Seongnam, Korea) [29]. Intra-assay coefficients of variations was <4.9% and inter-assay coefficients of variations was <9.0%. Mean values of duplicated assay were used for reporting results.

### 2.4. Determination of Long-Term Visit-to-Visit BPV

BP was measured by an electronic sphygmomanometer after seated rest for 5 min, at 0, 6, and 12 months and then yearly thereafter up to 8 years. Long-term visit-to-visit BPV was determined by average real variability (ARV), standard deviation (SD), and coefficient of variation (CoV) of systolic and diastolic BP (SBP and DBP) across visits. The median number of BP measurement in the study participants was 7 times.

### 2.5. Study Outcomes

The changes in ARV (in mmHg), SD (in mmHg), and CoV of SBP and DBP by serum OPG level (in pmol/L) were analyzed. ARV of SBP was used in the primary analysis, as its correlation with serum OPG level was strongest (Pearson r = 0.195, *p* < 0.001), while the others were used in the secondary analyses.

### 2.6. Statistical Analysis

Continuous variables were expressed as mean ± standard deviation or median [interquartile range]. Categorical variables were expressed as number of participants and percentage. For descriptive analyses, Student’s *t* test or one-way analysis of variance and χ2 test were used for continuous and categorical variates, respectively. The subjects with any missing data were excluded from further analyses. Multivariate linear regression analyses were used to define the association between serum OPG level and BPV. The models were adjusted for age, sex, Charlson comorbidity index, smoking history, BMI, SBP, DBP, medication (ACEi/ARB, diuretic use, number of antihypertensive drugs, statins), hemoglobin, albumin, HDL-C, fasting glucose, 25(OH) vitamin D, hs-CRP levels, eGFR and 24 h urine protein. The results of multivariate linear regression models were presented as beta coefficient and 95% CIs. Restricted cubic splines were used to visualize the association between serum OPG level as a continuous variable and adjusted beta coefficients for BPV indices. Two-sided *p* values <0.05 were considered statistically significant. Statistical analysis was performed using SPSS for Windows version 22.0 (IBM Corp., Armonk, NY, USA) and R (version 4.1.1; R project for Statistical Computing, Vienna, Austria).

## 3. Results

### 3.1. Baseline Characteristics

To address the baseline characteristics, study participants were divided into the quartile by serum OPG level (Table 1). The mean age was lowest and highest in the 1st (Q1) and 4th (Q4) quartile, respectively. Inversely, the frequency of male sex was highest in Q1. Most of the subjects in Q1 (94.8%) belonged to Charlson commodity index ≤3, while the frequency of the subjects with Charlson commodity index ≥4 was relatively higher in Q4. The frequency of the subjects with previous history of DM and coronary artery disease was significantly higher in Q4, while there was no significant difference in the frequency of the history of arrhythmia and ever-time smoking among the quartile groups. The proportion of the subjects with DM increased as serum OPG level increased, whereas the proportion of the subjects with glomerulonephritis or polycystic kidney disease decreased as serum OPG increased. Although the frequency of ACEi/ARB use was not significantly different among the groups, the use of diuretics, the use of no less than three antihypertensive drugs, and statin medication was more frequently observed in Q4. BMI did not significantly differ among the groups either. SBP gradually increases from Q1 to Q4, whereas DBP, despite the significant difference across the groups, did not show any clear correlation with serum OPG level. Hemoglobin and albumin levels were highest and lowest in Q1 and Q4, respectively. Total cholesterol, LDL-C, and TG levels were highest in Q4, whereas HDL-C level was lowest in Q4. Fasting glucose and hs-CRP levels were highest in Q4, while 25(OH) vitamin D level was lowest in Q4. Both proteinuria in 24 h urine and albumin-to-creatinine ratio in spot urine were highest in Q4. eGFR was also significantly lower in Q4. Accordingly, the subjects with more advanced stages of CKD were more frequently observed in Q4. Taken together, high serum OPG level was clearly associated with deleterious clinical features in patients with CKD.

### 3.2. Association of Serum OPG Level with BPV in Patients with CKD

**Figure 2:**
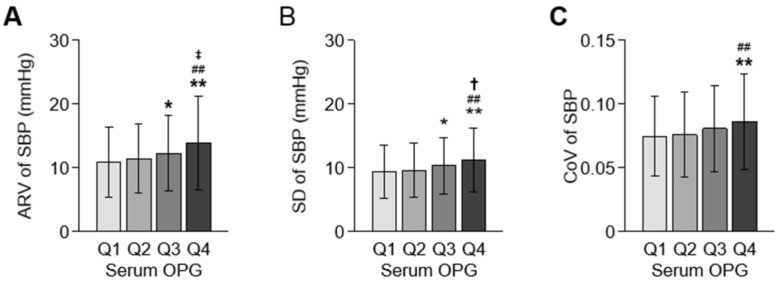
Comparisons of systolic BPV by serum OPG level. Systolic BPV, represented by ARV (**A**), SD (**B**), and CoV (**C**), is compared by the quartile of serum OPG level. Error bars indicate standard deviation. * *p* < 0.05, ** *p* < 0.01 vs. Q1; ^##^ *p* < 0.01 vs. Q2; ^†^ *p* < 0.05, ^‡^ *p* < 0.01 vs. Q3 by one-way ANOVA with Scheffe test. Abbreviations: ARV, average real variability; CoV, coefficient of variation; OPG, osteoprotegerin; Q1, 1st quartile; Q2, 2nd quartile; Q3, 3rd quartile; Q4, 4th quartile; SBP, systolic blood pressure; SD, standard deviation.

**Figure 3:**
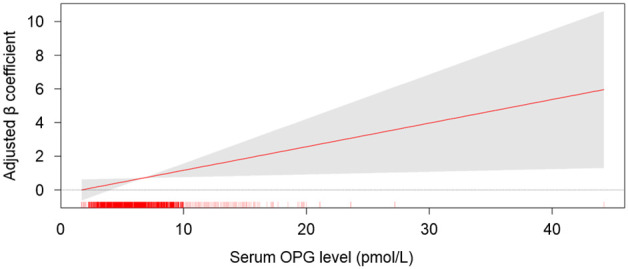
Restricted cubic spline of serum OPG on ARV of SBP. Adjusted β coefficient of serum OPG as a continuous variable for ARV of SBP is depicted. The model was adjusted for age, gender, Charlson comorbidity index, smoking history, BMI, SBP, DBP, medication (ACEi/ARBs, diuretics, number of antihypertensive drugs, statins), hemoglobin, albumin, HDL-C, fasting serum glucose, hs-CRP, 25(OH) vitamin D levels, eGFR, and 24 h urine protein. Abbreviations: OPG, osteoprotegerin.

To compare SBP variability among the quartile group by serum OPG levels, one-way analysis of variance was conducted (Figure 2). All of ARV, SD, and CoV of SBP significantly increased as serum OPG increased. ARV and CoV of DBP were also highest in Q4 (Figure S1). To determine the independent association between serum OPG level and systolic BPV, multivariate linear regression models were analyzed (Table 2). In the analyses of all subjects, ARV of SBP (Adjusted β coefficient 0.143, 95% confidence interval (CI) 0.021 to 0.264, *p* = 0.021) was significantly associated with serum OPG level. The association between serum OPG level and DBP variability was not significant (Table S1), regardless of BPV indices. To visualize the association between serum OPG level and SBP variability, restricted cubic splines were constructed, which revealed a stringent, linear correlation between serum OPG level and ARV of SBP (Figure 3). Although SD (Adjusted β coefficient 0.074, 95% CI -0.018 to 0.165, *p* = 0.113) or CoV of SBP (Adjusted β coefficient 0.000, 95% CI 0.000 to 0.000, *p* = 0.203) was not significantly associated with serum OPG level in the multivariate linear regression model (Table 2), restricted cubic splines demonstrated linear correlations (Figure S2).

### 3.3. Sensitivity Analysis

To substantiate our findings, we conducted sensitivity analyses by excluding the subjects with CKD stage 1 (*n* = 312), who are considered close to normal kidney function (Table 3). Multivariate linear regression analyses revealed that serum OPG level was robustly and significantly associated with ARV of SBP (Adjusted β coefficient 0.143, 95% CI 0.008 to 0.277, *p* = 0.038). In addition, to exclude those with possible risk of exaggerated BPV due to less frequent BP measurement, we only included the subjects with BP measurement no less than 5 time (*n* = 1502) and analyzed the association of serum OPG level and BPV. Despite the decreased number of the subjects being analyzed, serum OPG level was robustly and significantly associated with SD (Adjusted β coefficient 0.149, 95% CI 0.034 to 0.264, *p* = 0.011), and CoV (Adjusted β coefficient 0.001, 95% CI 0.000 to 0.002, *p* = 0.025), but not with ARV (Adjusted β coefficient 0.096, 95% CI -0.022 to 0.264, *p* = 0.096), of SBP (Table S2). Among the subjects with BP measurement no less than 5 time, serum OPG level was not significantly associated with any of ARV (Adjusted β coefficient 0.071, 95% CI -0.036 to 0.178, *p* = 0.196), SD (Adjusted β coefficient 0.072, 95% CI -0.010 to 0.155, *p* = 0.086), and CoV (Adjusted β coefficient 0.001, 95% CI 0.000 to 0.002, *p* = 0.055) of DBP (Table S3). Overall, the sensitivity analyses confirmed that serum OPG level is significantly associated with BPV in patients with pre-dialysis CKD.

### 3.4. Subgroup Analysis

To address whether the association of serum OPG level with BPV modified by clinical contexts, we conducted subgroup analyses. The subgroups were stratified by age (<60 or ≥60 years), gender (male or female), Charlson comorbidity index (≤3 or ≥4), BMI (<23 or ≥23 kg/m^2^), history of DM (without or with), eGFR (≥45 or <45 mL/min/1.73m^2^), 24 h urine protein (<200 or ≥200 mg/g). Although the association between serum OPG level and ARV of SBP was not significantly modified in any subgroups (Table 4), the association of serum OPG level with SD (Table 5) and CoV of SBP (Table S4) was significantly modified by Charlson comorbidity index and history of DM, suggesting that the association of serum OPG with BPV is more prominent in the subjects with Charlson comorbidity index ≤3 and in the subjects without history of DM. The association between serum OPG level and ARV, SD, or CoV of DBP was not significantly modified in any subgroups (Tables S5–S7).

## 4. Discussion

In the present study, we found a significant association between circulating OPG level and long-term visit-to-visit BPV in patients with pre-dialysis CKD. The association between circulating OPG level with BPV was especially prominent in the subjects with Charlson comorbidity index ≤3 and in the subjects without history of DM.

Elevation of serum OPG level in the subjects with reduced eGFR has been previously reported [30]. Despite the debate on the role of circulating OPG in atherosclerotic disease [31], as an experimental study reported that exogenous OPG treatment lead to endothelial and vascular smooth cell dysfunction by promoting the production of reactive oxygen species, which may underlie vascular injurious effects in conditions such as hypertension [32], the phenotype of OPG-knockout mice ultimately indicates circulating OPG as a biomarker, rather than a mediator, of atherosclerosis, as OPG-deficient mice develop early onset arterial calcification [33]. In this context, the association of high serum OPG level with coronary artery calcification [15,16], and cardiovascular [17,18] and all-cause mortality [19,20] in patients with CKD has been suggested. The association between serum OPG level with long-term visit-to-visit BPV presented in this study highlights a novel role of circulating OPG as a biomarker that predict a surrogate of CV events. It should be further elucidated whether the association is also valid in general population or in patients with end-stage renal disease.

We could not present a precise mechanism of the association between circulating OPG level and BPV, thought a possible explanation is an effect that is mediated by arterial stiffness. Mounting evidence suggests that serum OPG level is associated with vascular calcification and arterial stiffness as well as coronary artery calcification [10,34,35]. As arterial stiffness significantly correlates with increase in BPV [36], it is speculated that arterial stiffness may mediate the association between circulating OPG level and BPV.

The methods for the evaluation of visit-to-visit BPV is variable. SD is relatively easier and probably more practical but tends to correlate with the average of blood pressure measurements. Therefore, CoV, which is calculated by dividing the mean value by the SD, has been also used to determine visit-to-visit BPV [37]. ARV, which is defined as the average of the absolute differences of consecutive measurements, is a more reliable and sensitive representation of time series variability despite relatively low sampling frequency than SD [38], which is the reason why ARV was used as the primary analysis in the current study. The other index of BPV is the variation independent of the mean (VIM), which is calculated based on non-linear regression [39]. Although VIM is considered to be a better index of BP variability than the other indices, because VIM is literally not association with mean blood pressure. However, VIM was not evaluated in this study, as there is a significant difference between VIM and the other indices of BPV (SD, CoV, and ARV) and as it is less practical in clinical perspectives [37].

There are a number of limitations in this study. First, we did not analyze whether the casual relation between high circulating OPG and previously known adverse CV outcomes is mediated by high BPV. Second, despite the clear association of high circulating OPG with BPV, the precise mechanism should be further addressed. Third, although the results indicate an independent association of serum OPG level and BPV, a possibility cannot be still excluded that the factors other than BPV may have some effects on serum OPG levels, because the baseline characteristics were strikingly differed by serum OPG levels. Fourth, as this cohort study enrolled only ethnic Koreans, a precaution is required to extrapolate the data in the present study to other populations.

## 5. Conclusions

In conclusion, we report a potential association between circulating OPG level and long-term visit-to-visit BPV in patients with pre-dialysis CKD. The association between circulating OPG level with BPV is especially prominent in the subjects with Charlson comorbidity index ≤3 and in the subjects without history of DM.

## Figures and Tables

**Table 1:** Baseline characteristics of study participants by serum OPG level.

	Serum OPG Level				*p* Value
	Q1	Q2	Q3	Q4	
Age (Year)	43.796 ± 10.698	51.159 ± 10.435	56.517 ± 10.170	62.702 ± 7.903	<0.001
Male	300 (64.5)	365 (57.0)	262 (56.7)	284 (59.9)	0.044
Charlson comorbidity index					<0.001
0–3	411 (94.8)	391 (84.1)	366 (72.7)	195 (42.1)	
4–5	24 (5.2)	71 (15.3)	118 (25.5)	248 (53.6)	
6–7	0 (0.0)	3 (0.6)	8 (1.7)	19 (4.1)	
≥8	0 (0.0)	0 (0.0)	0 (0.0)	1 (0.2)	
DM	47 (10.1)	108 (23.2)	161 (34.8)	273 (59.0)	<0.001
CAD	3 (0.6)	20 (4.3)	26 (5.6)	57 (12.3)	<0.001
Arrhythmia	5 (1.1)	14 (3.0)	12 (2.6)	14 (3.0)	0.091
Primary renal disease					<0.001
DM	21 (4.5)	65 (14.0)	116 (25.1)	227 (49.0)	
HTN	75 (16.1)	95 (20.4)	110 (23.8)	95 (20.5)	
GN	225 (48.4)	181 (38.9)	140 (30.3)	63 (13.6)	
TID	4 (0.9)	1 (0.2)	4 (0.9)	5 (1.1)	
PKD	111 (23.9)	93 (20.0)	69 (14.9)	36 (7.8)	
Others	29 (6.2)	30 (6.5)	23 (5.0)	37 (8.0)	
Smoking history	249 (53.5)	260 (55.9)	256 (55.4)	239 (51.6)	0.44
Medication					
ACEi/ARBs	405 (87.1)	405 (87.1)	402 (87.0)	383 (82.7)	0.142
Diuretics	92 (19.8)	120 (25.8)	154 (33.3)	201 (43.4)	<0.001
Number of anti-HTN drugs ≥ 3	92 (19.8)	115 (24.7)	135 (29.2)	169 (36.5)	<0.001
Statins	189 (40.6)	233 (50.1)	271 (58.7)	266 (57.5)	<0.001
BMI (kg/m^2^)	24.486 ± 3.588	24.688 ± 3.332	24.626 ± 3.452	24.417 ± 3.092	0.595
SBP (mmHg)	124.232 ± 14.459	126.062 ± 14.2741	127.089 ± 15.269	129.827 ± 17.244	<0.001
DBP (mmHg)	77.531 ± 10.794	78.043 ± 10.429	76.513 ± 10.555	74.732 ± 11.772	<0.001
Laboratory findings					
Hemoglobin (g/dL)	13.800 ± 1.827	13.291 ± 1.896	12.870 ± 1.869	11.902 ± 1.803	<0.001
Albumin (g/dL)	4.303 ± 0.340	4.227 ± 0.368	4.204 ± 0.389	4.079 ± 0.407	<0.001
TC (mg/dL)	176.630 ± 32.653	176.561 ± 40.129	174.583 ± 41.353	168.070 ± 36.628	0.001
LDL-C (mg/dL)	99.008 ± 27.956	99.376 ± 33.178	95.311 ± 32.248	92.201 ± 28.663	0.001
HDL-C (mg/dL)	50.799 ± 15.382	51.306 ± 15.425	49.312 ± 15.425	47.376 ± 15.703	0.001
TG (mg/dL)	156.615 ± 98.887	145.480 ± 81.129	166.894 ± 113.306	156.892 ± 98.735	0.014
Fasting glucose (mg/dL)	101.280 ± 22.310	104.455 ± 28.829	112.650 ± 40.133	121.763 ± 53.667	<0.001
hsCRP (mg/dL)	0.450 (0.200, 1.200)	0.700 (0.300, 1.500)	0.635 ( 0.300, 1.600)	0.700 (0.200, 2.100)	0.019
25(OH) vitamin D (ng/dL)	18.570 ± 7.498	18.176 ± 6.907	17.971 ± 7.494	17.890 ± 9.306	0.559
24 h urine protein (mg/day)	368.000 (120.000, 888.000)	486.000 (138.000, 1330.000)	493.500 (191.925, 1274.125)	759.000 (280.000, 4205.000)	<0.001
Urine ACR (mg/g Cr)	196.928 (30.800, 565.480)	306.642 (55.529, 780.979)	314.765 (77.804, 863.397)	511.134 (144.603, 1490.550)	<0.001
eGFR (mL/min/1.73 m^2^)	68.717 ± 32.278	57.547 ± 30.177	47.749 ± 24.132	34.769 ± 18.409	<0.001
CKD stages					<0.001
Stage 1	157 (33.8)	94 (20.2)	47 (10.2)	14 (3.0)	
Stage 2	129 (27.7)	121 (26.0)	91 (19.7)	29 (6.3)	
Stage 3a	82 (17.6)	81 (17.4)	82 (19.9)	70 (15.1)	
Stage 3b	61 (13.1)	95 (20.4)	130 (28.1)	130 (28.1)	
Stage 4	33 (7.1)	63 (13.5)	89 (19.3)	183 (39.5)	
Stage 5	3 (0.6)	11 (2.4)	13 (2.8)	37 (8.0)	

Values for categorical variables are given as number (percentage); values for continuous variables, as mean ± standard deviation or median (interquartile range). Abbreviations: ACEi, angiotensin-converting enzyme inhibitor; ACR, albumin-to-creatinine ratio; ARB, angiotensin receptor blocker; BMI, body mass index; CAD, coronary artery disease; CCI, Charlson comorbidity index; CKD, chronic kidney disease; DBP, diastolic blood pressure; DM, diabetes mellitus; eGFR, estimated glomerular filtration rate; GN, glomerulonephritis; HDL-C, high density lipoprotein cholesterol; hs-CRP, high-sensitivity C-reactive protein; HTN, hypertension; LDL-C, low density lipoprotein cholesterol; OPG, osteoprotegerin; PKD, polycystic kidney disease; Q1, 1st quartile; Q2, 2nd quartile; Q3, 3rd quartile; Q4, 4th quartile; SBP, systolic blood pressure; TC, total cholesterol; TG, triglyceride; TID, tubulointerstitial disease.

**Table 2:** Multivariate linear regression analyses of serum OPG level (per pmol/L) for systolic BPV.

	Unadjusted		Adjusted	
	β Coefficient (95% CIs)	*p* Value	β Coefficient (95% CIs)	*p* Value
ARV of SBP	0.412 (0.320, 0.505)	<0.001	0.143 (0.021, 0.264)	0.021
SD of SBP	0.231 (0.162, 0.300)	<0.001	0.074 (−0.018, 0.165)	0.113
CoV of SBP	0.001 (0.001, 0.002)	<0.001	0.000 (0.000, 0.001)	0.203

Models were adjusted for age, gender, Charlson comorbidity index, smoking history, BMI, SBP, DBP, medication (ACEi/ARBs, diuretics, number of antihypertensive drugs, statins), hemoglobin, albumin, HDL-C, fasting serum glucose, hs-CRP, 25(OH) vitamin D levels, eGFR, and 24 h urine protein. Abbreviations: ARV, average real variability; CI, confidence interval; CoV, coefficient of variation; SBP, systolic blood pressure; SD, standard deviation.

**Table 3:** Multivariate linear regression analyses of serum OPG level (per pmol/L) for SBPV excluding the subjects with CKD stage 1.

	Unadjusted		Adjusted	
	β Coefficient (95% CIs)	*p* Value	β Coefficient (95% CIs)	*p* Value
ARV of SBP	0.373 (0.270, 0.476)	<0.001	0.143 (0.008, 0.277)	0.038
SD of SBP	0.200 (0.124, 0.275)	<0.001	0.081 (−0.018, 0.181)	0.107
CoV of SBP	0.001 (0.001, 0.002)	<0.001	0.001 (0.000, 0.001)	0.179

Models were adjusted for age, gender, Charlson comorbidity index, smoking history, BMI, SBP, DBP, medication (ACEi/ARBs, diuretics, number of antihypertensive drugs, statins), hemoglobin, albumin, HDL-C, fasting serum glucose, hs-CRP, 25(OH) vitamin D levels, eGFR, and 24 h urine protein. Abbreviations: ARV, average real variability; BMI, body mass index; CI, confidence interval; CoV, coefficient of variation; SBP, systolic blood pressure; SD, standard deviation.

**Table 4:** Multivariate linear regression analyses of serum OPG level (per pmol/L) for ARV of SBP in various subgroups.

	Unadjusted		Adjusted	
	β Coefficient (95% CIs)	*p* for Interaction	β Coefficient (95% CIs)	*p* for Interaction
Age < 60 years	0.499 (0.339, 0.658)	0.151	0.018 (−0.179, 0.215)	0.131
Age ≥ 60 years	0.377 (0.227, 0.528)		0.171 (−0.009, 0.352)	
Male	0.480 (0.354, 0.606)	0.955	0.224 (0.050, 0.399)	0.465
Female	0.321 (0.184, 0.457)		0.046 (−0.127, 0.220)	
Charlson comorbidity index ≤ 3	0.361 (0.239, 0.484)	0.03	0.143 (−0.008, 0.294)	0.165
Charlson comorbidity index ≥ 4	0.162 (−0.025, 0.348)		0.174 (−0.060, 0.407)	
History of DM (−)	0.403 (0.265, 0.541)	0.055	0.153 (−0.027, 0.333)	0.228
History of DM (+)	0.218 (0.066, 0.370)		0.185 (−0.003, 0.373)	
BMI < 23 kg/m^2^	0.447 (0.279, 0.615)	0.739	0.100 (−0.146, 0.347)	0.801
BMI ≥ 23 kg/m^2^	0.396 (0.284, 0.507)		0.177 (0.037, 0.318)	
Number of anti-HTN drugs ≤ 2	0.399 (0.291, 0.507)	0.777	0.112 (−0.029, 0.253)	0.871
Number of anti-HTN drugs ≥ 3	0.377 (0.192, 0.562)		0.194 (−0.050, 0.438)	
eGFR ≥ 45 mL/min/1.73 m^2^	0.418 (0.255, 0.581)	0.986	0.172 (−0.062, 0.347)	0.281
eGFR < 45 mL/min/1.73 m^2^	0.339 (0.201, 0.477)		0.106 (−0.073, 0.284)	
24 h urine protein < 200 mg	0.530 (0.340, 0.720)	0.108	0.366 (0.124, 0.607)	0.149
24 h urine protein ≥ 200 mg	0.363 (0.253, 0.472)		0.103 (−0.040, 0.246)	

Models were adjusted for age, gender, Charlson comorbidity index, smoking history, BMI, SBP, DBP, medication (ACEi/ARBs, diuretics, number of antihypertensive drugs, statins), hemoglobin, albumin, HDL-C, fasting serum glucose, hs-CRP, 25(OH) vitamin D levels, eGFR, and 24 h urine protein. Abbreviations: CI, confidence interval; DM, diabetes mellitus; eGFR, estimated glomerular filtration rate; HTN, hypertension.

**Table 5:** Multivariate linear regression analyses of serum OPG level (per pmol/L) for SD of SBP in various subgroups.

	Unadjusted		Adjusted	
	β Coefficient (95% CIs)	*p* for Interaction	β Coefficient (95% CIs)	*p* for Interaction
Age < 60 years	0.272 (0.151, 0.393)	0.040	−0.001 (−0.153, 0.150)	0.388
Age ≥ 60 years	0.167 (0.059, 0.275)		0.095 (−0.036, 0.225)	
Male	0.258 (0.168, 0.348)	0.800	0.142 (0.016, 0.268)	0.630
Female	0.195 (0.089, 0.302)		−0.007 (−0.144, 0.130)	
Charlson comorbidity index ≤ 3	0.261 (0.167, 0.355)	<0.001	0.127 (0.009, 0.245)	0.005
Charlson comorbidity index ≥ 4	0.003 (−0.127, 0.133)		0.062 (−0.100, 0.225)	
History of DM (−)	0.263 (0.158, 0.369)	0.007	0.145 (0.006, 0.284)	0.043
History of DM (+)	0.067 (−0.041, 0.175)		0.051 (−0.084, 0.186)	
BMI < 23 kg/m^2^	0.237 (0.112, 0.362)	0.998	−0.007 (−0.193, 0.178)	0.868
BMI ≥ 23 kg/m^2^	0.228 (0.146, 0.311)		0.118 (0.013, 0.224)	
Number of anti-HTN drugs ≤ 2	0.229 (0.149, 0.310)	0.411	0.057 (−0.049, 0.163)	0.973
Number of anti-HTN drugs ≥ 3	0.204 (0.067, 0.341)		0.105 (−0.074, 0.285)	
eGFR ≥ 45 mL/min/1.73 m^2^	0.355 (0.228, 0.483)	0.033	0.145 (−0.016, 0.305)	0.421
eGFR < 45 mL/min/1.73 m^2^	0.171 (0.073, 0.270)		0.074 (−0.055, 0.203)	
24 h urine protein < 200 mg	0.354 (0.213, 0.496)	0.055	0.194 (0.011, 0.377)	0.103
24 h urine protein ≥ 200 mg	0.186 (0.105, 0.267)		0.060 (−0.048, 0.167)	

Models were adjusted for age, gender, Charlson comorbidity index, smoking history, BMI, SBP, DBP, medication (ACEi/ARBs, diuretics, number of antihypertensive drugs, statins), hemoglobin, albumin, HDL-C, fasting serum glucose, hs-CRP, 25(OH) vitamin D levels, eGFR, and 24 h urine protein. Abbreviations: CI, confidence interval; DM, diabetes mellitus; eGFR, estimated glomerular filtration rate; HTN, hypertension.

## Data Availability

The raw data supporting the conclusions of this article will be made available by the authors, without undue reservation.

## Supplementary Materials

The following are available online at https://www.mdpi.com/article/10.3390/jcm11010178/s1, Figure S1: Restricted cubic spline of serum OPG on SD and CoV of SBP, Table S1: Multivariate linear regression analyses of serum OPG level (per pmol/L) for DBPV, Table S2: Multivariate linear regression analyses of serum OPG level (per pmol/L) for SBPV in subjects with BP measurements no less than 5 times during follow-up periods, Table S3: Multivariate linear regression analyses of serum OPG level (per pmol/L) for DBPV in subjects with BP measurements no less than 5 times during follow-up periods, Table S4: Multivariate linear regression analyses of serum OPG level (per pmol/L) for ARV of SBP in various subgroups, Table S5: Multivariate linear regression analyses of serum OPG level (per pmol/L) for CoV of SBP in various subgroups, Table S6: Multivariate linear regression analyses of serum OPG level (per pmol/L) for ARV of DBP in various subgroups.
