# Supplementary material for: Association of Circulating Osteoprotegerin Level with Blood Pressure Variability in Patients with Chronic Kidney Disease
Source: J Clin Med. 2021 Dec 29;11(1):178. doi: 10.3390/jcm11010178 (PMC8745733; doi:10.3390/jcm11010178)
Supplement: Supplementary file 1 [file jcm-11-00178-s001.zip › jcm-1436836-supplementary.pdf]

Supplementary Material

Association of circulating osteoprotegerin level with blood pressure variability in patients with chronic kidney disease

Sang Heon Suh[1], Tae Ryom Oh[1], Hong Sang Choi[1], Chang Seong Kim[1], Kook-Hwan Oh[2], Joongyub Lee[3], Yun Kyu Oh[4], Ji Yong Jung[5], Kyu Hun Choi[6], Seong Kwon Ma[1], Eun Hui Bae[1]*, and Soo Wan Kim[1]*, on behalf of the Korean Cohort Study for Outcomes in Patients With Chronic Kidney Disease (KNOW-CKD) Investigators

[1]Department of Internal Medicine, Chonnam National University Medical School, Gwangju, Korea
[2]Department of Internal Medicine, Seoul National University Hospital, Seoul, Korea
[3]Department of Prevention and Management, School of Medicine, Inha University, Incheon, Republic of Korea
[4]Department of Internal Medicine, Seoul National University, Seoul, Korea
[5]Division of Nephrology, Department of Internal Medicine, Gachon University of Gil Medical Center, Incheon, Republic of Korea
[6]Department of Internal Medicine, College of Medicine, Institute of Kidney Disease Research, Yonsei University, Seoul, Korea

Corresponding authors

*Eun Hui Bae, M.D., Ph.D., Department of Internal Medicine, Chonnam National University Medical School, 42 Jebongro, Gwangju 61469, Korea, Tel: +82-62-220-6503, Fax: +82-62-225-8578, Email: baedak76@gmail.com

*Soo Wan Kim, M.D., Ph.D., Department of Internal Medicine, Chonnam National University Medical School, 42 Jebongro, Gwangju 61469, Korea, Tel: +82-62-220-6271, Fax: +82-62-225-8578, Email: skimw@chonnam.ac.kr

<u>Table of contents</u>

Figure S1. Comparisons of diastolic BPV by serum OPG level
Figure S2. Restricted cubic spline of serum OPG on SD and CoV of SBP
Table S1. Multivariate linear regression analyses of serum OPG level (per pmol/L) for Diastolic BPV
Table S2. Multivariate linear regression analyses of serum OPG level (per pmol/L) for Systolic BPV in subjects with BP measurements no less than 5 times during follow-up periods
Table S3. Multivariate linear regression analyses of serum OPG level (per pmol/L) for diastolic BPV in subjects with BP measurements no less than 5 times during follow-up periods
Table S4. Multivariate linear regression analyses of serum OPG level (per pmol/L) for CoV of SBP in various subgroups
Table S5. Multivariate linear regression analyses of serum OPG level (per pmol/L) for ARV of DBP in various subgroups
Table S6. Multivariate linear regression analyses of serum OPG level (per pmol/L) for SD of DBP in various subgroups
Table S7. Multivariate linear regression analyses of serum OPG level (per pmol/L) for CoV of DBP in various subgroups

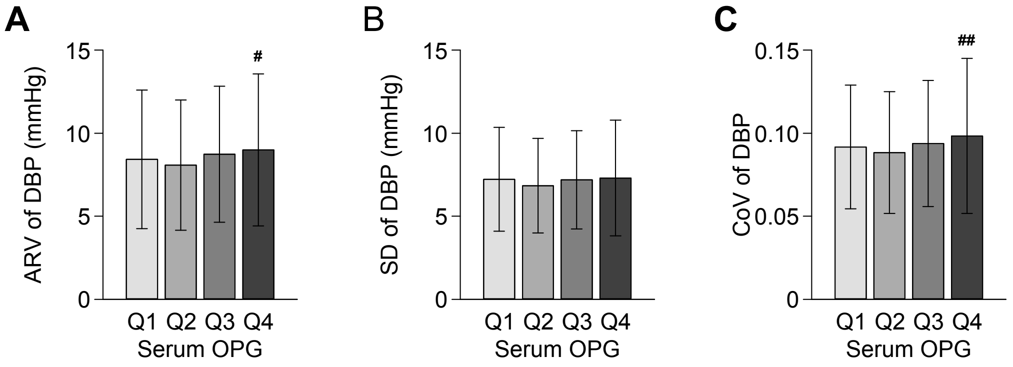

Figure S1. Comparisons of diastolic BPV by serum OPG level

Note: Diastolic BPV, represented by ARV (A), SD (B), and CoV (C), is compared by the quartile of serum OPG level. Error bars indicate standard deviation. **P* < 0.05, ***P* < 0.01 vs. Q1; ##*P* < 0.01 vs. Q2; †*P* <0.05, ‡*P* <0.01 vs. Q3 by one-way ANOVA with Scheffe test. Abbreviations: ARV, average real variability; CoV, coefficient of variation; OPG, osteoprotegerin; Q1, 1st quartile; Q2, 2nd quartile; Q3, 3rd quartile; Q4, 4th quartile; SBP, systolic blood pressure; SD, standard deviation.

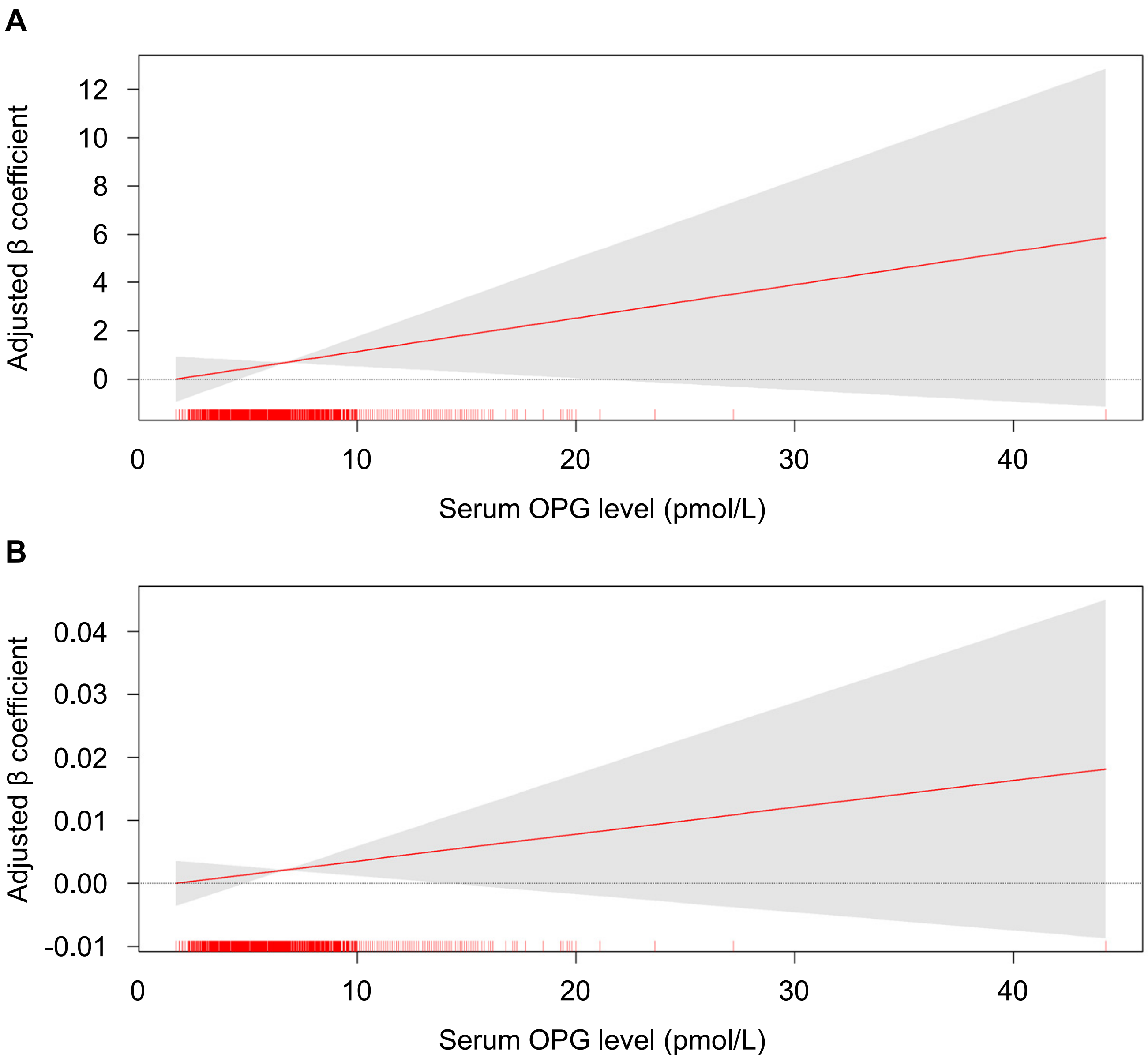

Figure S2. Restricted cubic spline of serum OPG on SD and CoV of SBP

Note: Adjusted β coefficient of serum OPG as a continuous variable for SD (A) and CoV (B) of SBP is depicted. The models were adjusted for age, gender, Charlson comorbidity index, smoking history, BMI, SBP, DBP, medication (ACEi/ARBs, diuretics, number of antihypertensive drugs, statins), hemoglobin, albumin, HDL-C, fasting serum glucose, hs-CRP, 25(OH) vitamin D levels, eGFR, and 24-hour urine protein. Abbreviations: OPG, osteoprotegerin.

Table S1. Multivariate linear regression analyses of serum OPG level (*per* pmol/L) for diastolic BPV

	Unadjusted		Adjusted	
	β coefficient (95%CIs)	*P* value	β coefficient (95%CIs)	*P* value
ARV of DBP	0.070 (0.006, 0.134)	0.033	0.017 (-0.068, 0.102)	0.696
SD of DBP	0.010 (-0.038, 0.057)	0.685	0.020 (-0.044, 0.083)	0.547
CoV of DBP	0.001 (0.000, 0.001)	0.019	0.000 (0.000, 0.001)	0.438

Note: Models were adjusted for age, gender, Charlson comorbidity index, smoking history, BMI, SBP, DBP, medication (ACEi/ARBs, diuretics, number of antihypertensive drugs, statins), hemoglobin, albumin, HDL-C, fasting serum glucose, hs-CRP, 25(OH) vitamin D levels, eGFR, and 24-hour urine protein.

Abbreviations: ARV, average real variability; CI, confidence interval; CoV, coefficient of variation; DBP, diastolic blood pressure; OPG, osteoprotegerin; SD; standard deviation.

Table S2. Multivariate linear regression analyses of serum OPG level (*per* pmol/L) for systolic BPV in subjects with BP measurements no less than 5 times during follow-up periods

	Unadjusted		Adjusted	
	β coefficient (95%CIs)	*P* value	β coefficient (95%CIs)	*P* value
ARV of SBP	0.367 (0.258, 0.475)	< 0.001	0.121 (-0.022, 0.264)	0.096
SD of SBP	0.317 (0.231, 0.404)	< 0.001	0.149 (0.034, 0.264)	0.011
CoV of SBP	0.002 (0.001, 0.003)	< 0.001	0.001 (0.000, 0.002)	0.025

Note: Models were adjusted for age, gender, Charlson comorbidity index, smoking history, BMI, SBP, DBP, medication (ACEi/ARBs, diuretics, number of antihypertensive drugs, statins), hemoglobin, albumin, HDL-C, fasting serum glucose, hs-CRP, 25(OH) vitamin D levels, eGFR, and 24-hour urine protein.
Abbreviations: ARV, average real variability; CI, confidene interval; CoV, coefficient of variation; SBP, systolic blood pressure; SD; standard deviation.

Table S3. Multivariate linear regression analyses of serum OPG level (*per* pmol/L) for diastolic BPV in subjects with BP measurements no less than 5 times during follow-up periods

	Unadjusted		Adjusted	
	β coefficient (95%CIs)	*P* value	β coefficient (95%CIs)	*P* value
ARV of DBP	0.106 (0.026, 0.186)	0.009	0.071 (-0.036, 0.178)	0.193
SD of DBP	0.060 (-0.001, 0.122)	0.054	0.072 (-0.010, 0.155)	0.086
CoV of DBP	0.002 (0.001, 0.002)	< 0.001	0.001 (0.000, 0.002)	0.055

Note: Models were adjusted for age, gender, Charlson comorbidity index, smoking history, BMI, SBP, DBP, medications (ACEi/ARBs, diuretics, number of antihypertensive drugs, statins), hemoglobin, albumin, HDL-C, fasting serum glucose, hs-CRP, 25(OH) vitamin D levels, eGFR, and 24-hour urine protein. Abbreviations: ARV, average real variability; CI, confidence interval; CoV, coefficient of variation; DBP, diastolic blood pressure; SD; standard deviation.

Table S4. Multivariate linear regression analyses of serum OPG level (*per* pmol/L) for CoV of SBP in various subgroups

	Unadjusted		Adjusted	
	β coefficient (95%CIs)	*P* for interaction	β coefficient (95%CIs)	*P* for interaction
Age < 60 years	0.002 (0.001, 0.003)	0.035	0.000 (-0.001, 0.001)	0.492
Age ≥ 60 years	0.001 (0.000, 0.002)		0.001 (0.000, 0.002)	
Male	0.002 (0.001, 0.002)	0.687	0.001 (0.000, 0.002)	0.485
Female	0.001 (0.000, 0.002)		0.000 (-0.001, 0.001)	
CCI ≤ 3	0.002 (0.001, 0.003)	< 0.001	0.001 (0.000, 0.002)	0.002
CCI ≥ 4	0.000 (-0.001, 0.001)		0.000 (-0.001, 0.002)	
History of DM (-)	0.002 (0.001, 0.003)	0.006	0.001 (0.000, 0.002)	0.04
History of DM (+)	0.000 (-0.001, 0.001)		0.000 (-0.001, 0.001)	
BMI < 23 kg/m^2	0.001 (0.000, 0.002)	0.605	0.000 (-0.002, 0.001)	0.633
BMI ≥ 23 kg/m^2	0.002 (0.001, 0.002)		0.001 (0.000, 0.002)	
Number of anti-HTN drugs ≤2	0.002 (0.001, 0.002)	0.327	0.000 (0.000, 0.001)	0.765
Number of anti-HTN drugs ≥ 3	0.001 (0.000, 0.002)		0.001 (-0.001, 0.002)	
eGFR ≥ 45 mL/min./1.73m^2	0.002 (0.002, 0.003)	0.019	0.001 (0.000, 0.002)	0.234
eGFR < 45 mL/min./1.73m^2	0.001 (0.000, 0.002)		0.000 (-0.001, 0.001)	
24-hour urine protein < 200 mg	0.003 (0.002, 0.004)	0.028	0.001 (0.000, 0.003)	0.066
24-hour urine protein ≥ 200 mg	0.001 (0.000, 0.002)		0.000 (0.000, 0.001)	

Note: Models were adjusted for age, gender, Charlson comorbidity index, smoking history, BMI, SBP, DBP, medication (ACEi/ARBs, diuretics, number of antihypertensive drugs, statins), hemoglobin, albumin, HDL-C, fasting serum glucose, hs-CRP, 25(OH) vitamin D levels, eGFR, and 24-hour urine protein. Abbreviations: CI, confidence interval; DM, diabetes mellitus; eGFR, estimated glomerular filtration rate; HTN, hypertension.

Table S5. Multivariate linear regression analyses of serum OPG level (*per* pmol/L) for ARV of DBP in various subgroups

	Unadjusted		Adjusted	
	β coefficient (95%CIs)	*P* for interaction	β coefficient (95%CIs)	*P* for interaction
Age < 60 years	0.168 (0.051, 0.284)	0.120	-0.012 (-0.159, 0.134)	0.933
Age ≥ 60 years	0.071 (-0.024, 0.165)		0.051 (-0.064, 0.165)	
Male	0.086 (0.002, 0.171)	0.887	0.077 (-0.041, 0.196)	0.616
Female	0.048 (-0.050, 0.145)		-0.051 (-0.177, 0.076)	
CCI ≤ 3	0.051 (-0.038, 0.140)	0.690	0.040 (-0.072, 0.152)	0.468
CCI ≥ 4	-0.002 (-0.122, 0.117)		-0.021 (-0.172, 0.130)	
History of DM (-)	0.021 (-0.077, 0.120)	0.918	0.049 (-0.081, 0.180)	0.743
History of DM (+)	0.030 (-0.071, 0.132)		-0.022 (-0.148, 0.104)	
BMI < 23 kg/m^2	0.034 (-0.081, 0.149)	0.931	-0.070 (-0.241, 0.101)	0.408
BMI ≥ 23 kg/m^2	0.086 (0.009, 0.163)		0.056 (-0.043, 0.156)	
Number of anti-HTN drugs ≤2	0.060 (-0.013, 0.134)	0.616	-0.006 (-0.104, 0.901)	0.934
Number of anti-HTN drugs ≥ 3	0.046 (-0.086, 0.178)		0.080 (-0.093, 0.253)	
eGFR ≥ 45 mL/min./1.73m^2	0.060 (-0.061, 0.182)	0.874	0.054 (-0.101, 0.210)	0.803
eGFR < 45 mL/min./1.73m^2	0.041 (-0.049, 0.131)		0.028 (-0.089, 0.145)	
24-hour urine protein < 200 mg	0.045 (-0.099, 0.188)	0.619	0.085 (-0.108, 0.278)	0.843
24-hour urine protein ≥ 200 mg	0.060 (-0.014, 0.133)		0.003 (-0.094, 0.100)	

Note: Models were adjusted for age, gender, Charlson comorbidity index, smoking history, BMI, SBP, DBP, medication (ACEi/ARBs, diuretics, number of antihypertensive drugs, statins), hemoglobin, albumin, HDL-C, fasting serum glucose, hs-CRP, 25(OH) vitamin D levels, eGFR, and 24-hour urine protein. Abbreviations: CI, confidence interval; DM, diabetes mellitus; eGFR, estimated glomerular filtration rate; HTN, hypertension.

Table S6. Multivariate linear regression analyses of serum OPG level (*per* pmol/L) for SD of DBP in various subgroups

	Unadjusted		Adjusted	
	β coefficient (95%CIs)	*P* for interaction	β coefficient (95%CIs)	*P* for interaction
Age < 60 years	0.070 (-0.008, 0.148)	0.274	0.011 (-0.099, 0.120)	0.762
Age ≥ 60 years	0.015 (-0.048, 0.077)		0.033 (-0.054, 0.119)	
Male	0.019 (-0.008, 0.075)	0.0717	0.096 (0.007, 0.186)	0.278
Female	0.003 (-0.063, 0.068)		-0.080 (-0.174, 0.014)	
CCI ≤ 3	0.001 (-0.064, 0.065)	0.860	0.027 (-0.059, 0.113)	0.610
CCI ≥ 4	-0.008 (-0.080, 0.064)		0.027 (-0.079, 0.133)	
History of DM (-)	-0.011 (-0.081, 0.060)	0.996	0.046 (-0.055, 0.146)	0.913
History of DM (+)	-0.011 (-0.074, 0.053)		-0.012 (-0.102, 0.078)	
BMI < 23 kg/m^2	0.024 (-0.055, 0.103)	0.734	-0.057 (-0.187, 0.072)	0.637
BMI ≥ 23 kg/m^2	0.008 (-0.043, 0.059)		0.047 (-0.028, 0.121)	
Number of anti-HTN drugs ≤2	0.015 (-0.033, 0.064)	0.445	-0.014 (-0.086, 0.058)	0.728
Number of anti-HTN drugs ≥ 3	-0.021 (-0.113, 0.070)		0.098 (-0.037, 0.233)	
eGFR ≥ 45 mL/min./1.73m^2	0.056 (-0.033, 0.146)	0.039	0.089 (-0.033, 0.211)	0.496
eGFR < 45 mL/min./1.73m^2	0.003 (-0.053, 0.059)		0.035 (-0.049, 0.119)	
24-hour urine protein < 200 mg	-0.005 (-0.106, 0.096)	0.852	0.039 (-0.101, 0.178)	0.735
24-hour urine protein ≥ 200 mg	0.006 (-0.046, 0.058)		0.017 (-0.056, 0.091)	

Note: Models were adjusted for age, gender, Charlson comorbidity index, smoking history, BMI, SBP, DBP, medication (ACEi/ARBs, diuretics, number of antihypertensive drugs, statins), hemoglobin, albumin, HDL-C, fasting serum glucose, hs-CRP, 25(OH) vitamin D levels, eGFR, and 24-hour urine protein. Abbreviations: CI, confidence interval; DM, diabetes mellitus; eGFR, estimated glomerular filtration rate; HTN, hypertension.

Table S7. Multivariate linear regression analyses of serum OPG level (*per* pmol/L) for CoV of DBP in various subgroups

	Unadjusted		Adjusted	
	β coefficient (95%CIs)	*P* for interaction	β coefficient (95%CIs)	*P* for interaction
Age < 60 years	0.001 (0.000, 0.002)	0.221	0.000 (-0.001, 0.002)	0.825
Age ≥ 60 years	0.000 (0.000, 0.001)		0.000 (-0.001, 0.002)	
Male	0.000 (0.000, 0.001)	0.226	-0.001 (-0.002, 0.000)	0.167
Female	0.001 (0.000, 0.002)		0.001 (0.000, 0.003)	
CCI ≤ 3	0.000 (0.000, 0.001)	0.699	0.000 (-0.001, 0.002)	0.572
CCI ≥ 4	0.000 (-0.001, 0.001)		0.000 (-0.001, 0.002)	
History of DM (-)	0.000 (-0.001, 0.001)	0.964	0.001 (-0.001, 0.002)	0.849
History of DM (+)	0.000 (-0.001, 0.001)		0.000 (-0.001, 0.001)	
BMI < 23 kg/m^2	0.001 (0.000, 0.002)	0.919	-0.001 (-0.002, 0.001)	0.457
BMI ≥ 23 kg/m^2	0.001 (0.000, 0.001)		0.001 (0.000, 0.002)	
Number of anti-HTN drugs ≤2	0.001 (0.000, 0.001)	0.683	0.000 (-0.001, 0.001)	0.675
Number of anti-HTN drugs ≥ 3	0.001 (-0.001, 0.002)		0.001 (0.000, 0.003)	
eGFR ≥ 45 mL/min./1.73m^2	0.001 (0.000, 0.003)	0.194	0.001 (0.000, 0.003)	0.363
eGFR < 45 mL/min./1.73m^2	0.001 (0.000, 0.001)		0.000 (-0.001, 0.002)	
24-hour urine protein < 200 mg	0.001 (-0.001, 0.002)	0.885	0.000 (-0.001, 0.002)	0.698
24-hour urine protein ≥ 200 mg	0.001 (0.000, 0.001)		0.000 (-0.001, 0.001)	

Note: Models were adjusted for age, gender, Charlson comorbidity index, smoking history, BMI, SBP, DBP, medication (ACEi/ARBs, diuretics, number of antihypertensive drugs, statins), hemoglobin, albumin, HDL-C, fasting serum glucose, hs-CRP, 25(OH) vitamin D levels, eGFR, and 24-hour urine protein. Abbreviations: CI, confidence interval; DM, diabetes mellitus; eGFR, estimated glomerular filtration rate; HTN, hypertension.
